# Is the Zika virus re-emerging as a distinct genetic lineage in India?

**DOI:** 10.1099/acmi.0.000857.v4

**Published:** 2025-05-14

**Authors:** Pradeep Kumar N., Ajithlal P.M., Prasanta Saini, Aiswarya R. S., Abidha Suresh, Philip Samuel, Balasubramaniam R., Jessu Mathew, Sonia T., Amju K.P., Raju K. H. K., Veerapathiran A., Selvam A., Balaji T., Ashwani Kumar

**Affiliations:** 1 ICMR-Vector Control Research Centre, Field Station, Kottayam, Kerala, India; 2 ICMR-Vector Control Research Centre, Indira Nagar, Puducherry, India; 3 ICMR-Vector Control Research Centre, Field Station, Madurai, India; 4 ICMR-National Institute of Virology, Field Unit, Alappuzha, India

**Keywords:** Asian lineage, India, re-emerging diseases, ZIKV

## Abstract

An outbreak of Zika fever occurred in Thiruvananthapuram City, Kerala, India, during 2021. At the request of the Kerala state health administration, we investigated the same, towards proposing requisite containment strategies for the disease outbreak. Epidemiological investigations indicated a clustering pattern of Zika fever cases with the presumed index case from a multi-speciality hospital in the city. Preliminary reports on the same had been already reported elsewhere during 2021. Further, entomological surveys carried out evinced the predominant mosquito species in the city, viz. *Aedes albopictus* (65.55%), *Aedes aegypti* (22.0%) and *Aedes vittatus* (12.0%) were naturally infected with Zika virus (ZIKV), the minimum infection rates being 17.9, 7.8 and 3.6, respectively. Also, trans-ovarian transmission was recorded in both *Ae. aegypti* and *Ae. albopictus*. This is the first report on the detection of ZIKV from *Ae. albopictus* in India. Analysis of phylogenetically informative genes of the ZIKV genome indicated the emergence of a distinct lineage of the Asian strain of virus, with five unique non-synonymous mutations, viz. ‘A22T’ and ‘I160M’ (pre-membrane) and ‘D348N’, ‘T470A’ and ‘V473L’ (envelope), that were involved in the outbreak. The altered gene expression pattern and evolutionary implications of these unique mutations remain to be investigated. Genetic analysis of the virus isolates from this and other investigations carried out on sporadic outbreaks of ZIKV in the country subsequently indicated that ZIKV is re-emerging as a distinct genetic lineage in India. These findings and other recent reports on ZIKV outbreaks warrant an urgent need for a systematic countrywide surveillance strategy, towards the prevention/preparedness/containment of a massive outbreak of this emerging neurovirulent arboviral disease.

**Impact Statement**

This report describes the outcome of the epidemiological investigations carried out, which enabled the successful containment of the Zika virus (ZIKV) outbreak in Kerala State, India, during 2021. Our very comprehensive investigations discerned that ZIKV was transmitted by the *Aedes* species prevalent in the region, such as *Aedes albopictus*, *Aedes aegypti* and *Aedes vittatus*, during the outbreak. Also, ZIKV was detected from adult unfed females and males from both field-collected and freshly emerged specimens of *Ae. aegypti* and *Ae. albopictus* specimens, indicating trans-ovarian transmission of this virus in these species. Genetic analysis of ZIKV isolates from both humans as well as the vector species revealed five distinct non-synonymous mutations: ‘A22T’ and ‘I160M’ (pre-membrane) and ‘D348N’, ‘T470A’ and ‘V473L’ (envelope). These unique mutations indicated the emergence of a novel genetic lineage of the Asian strain of the virus, the ‘Indian lineage’, during the 2021 outbreak, in India. Some of these mutations have also been reported in recent outbreaks in India. We propose this emerging ‘Indian lineage’ evolving as the dominant strain in the country, which could potentially lead to unprecedented outbreaks in the future. Further research investigations are required to explore the altered gene expression patterns owing to these mutations in ZIKV and their evolutionary implications.

## Data summary

- **Diagnostic marker sequences**

1. Human sample 1: MZ670001
2. Human sample 2: MZ686204
3. One pool of *Ae. aegypti* (male) (ZT248): OP947010
4. One pool of *Ae. albopictus* (emerged males) (ZTE 25): OP947011
5. One pool of *Ae. albopictus* (male) (ZT35): MZ 686203
6. One pool of *Ae. albopictus* (female - unfed) (ZT32): MZ686202

- **Capsid–pre-membrane gene**

1. Human sample: OP679002
2. *Ae. albopictus*: OP679003

- **Envelope gene**

1. Human sample: OP679000
2. *Ae. albopictus*: OP679001

- **Non-structural protein 5 gene**

1. Human sample: OP678998
2. *Ae. albopictus*: OP678999

## Introduction

Zika fever caused by the Zika virus (ZIKV) (*Orthoflavivirus zikaense*) grabbed public health attention owing to the widespread global outbreak caused by it during 2015–2016. The genus *Orthoflavivirus* under the family *Flaviviridae* includes a group of positive-sense single-strand RNA viruses such as dengue virus, ZIKV, West Nile virus, yellow fever virus, Japanese encephalitis virus and Kyasanur Forest disease virus, which could cause tremendous human morbidity and mortality. The diseases caused by these viruses are classified as emerging/re-emerging diseases by the World Health Organization (WHO). ZIKV was first reported from rhesus monkeys belonging to the Zika Forest in Uganda in 1947. In 1952, the virus was found to be infecting human populations from Uganda and Tanzania [1]. From the 1960s to the 1980s, sporadic human infections with the virus causing mild fever were reported in Asian and African countries. The first reported outbreak of the disease in humans was recorded from Yap Island, Federated States of Micronesia, in 2007 [2]. This was followed by a larger one in French Polynesia in 2013 and other countries and territories in the Pacific [3]. However, the disease manifestations exhibited were only mild fever and were generally neglected. The disease spread to New Caledonia and Easter Island in the Pacific in 2014. Subsequently, it emerged as a major outbreak, with its invasion to Mexico, Central America, the Caribbean and South America. By 2016, the disease spread to 86 countries. Approximately 1.3 million people were affected, and the disease emerged as a more virulent form causing congenital Zika syndrome amongst neonates, whose mothers were infected by ZIKV during pregnancy. About 4,000 cases of congenital microcephaly amongst neonates were reported in South America [4]. Another severe disease manifestation caused by the infections was Guillain–Barré syndrome. Owing to the alarming spread of the disease and the severity of the newly emerged genotype of the virus, the WHO alerted all the member countries, declaring the Zika fever outbreak as a Public Health Emergency of International Concern (PHEIC) in January 2016, calling all of them to carry out intensive surveillance programmes as a preparedness measure to contain outbreaks.

The major vector species involved in ZIKV transmission is *Aedes aegypti*. In some countries (Gabon), *Aedes albopictus* was reported as playing a major role in the transmission of the diseases [5]. In addition to vector-driven human-to-human transmission, other routes of transmission such as congenital transmission, blood transfusion and sexual transmission have been recorded [6]. Two principal genotypes of ZIKV have been reported prior to the 2015 major outbreak of the virus. The prototypes of these two genotypes, viz. African and Asian, were (i) the isolate reported from the Zika Forest and (ii) the Malaysian isolate reported in 1966 from Bentong, Malaysia [7], respectively. Subsequently, the Asian genotype evolved into two lineages, viz. (i) the original one (Asian lineage) and (ii) the American lineage. The latter lineage led to the PHEIC, with its epicentre of infection in Bahia Province, Brazil [8].

In India, until 2016, only reports on antigenemia to ZIKV had been reported, from the central region of the country, viz. Maharashtra, Rajasthan and Gujarat. As immunological tests may cross-react with other flaviviruses, these cannot be confirmatory on the prevalence of ZIKV in the country. During 2017, sporadic infections of the disease were reported in Tamil Nadu and Gujarat. However, the first confirmed minor outbreak of ZIKV occurred in Rajasthan State during 2018, affecting about 159 people [9]. With limited genetic data available [10], the strain of the circulating virus was ascertained to be Asian [11].

In July 2021, an outbreak of Zika fever was recorded in Thiruvananthapuram, the capital city of Kerala State, located in South India. We carried out a systematic investigation on the epidemiological and entomological factors, at the request of the state health authorities, with the objective of devising a strategy towards containment of the outbreak. The first case and about 13 cases reported prior to that (which were confirmed retrospectively) were recorded from a multi-speciality hospital located in the western central region of the capital city.

## Methods

### Study area

**Fig. 1.**
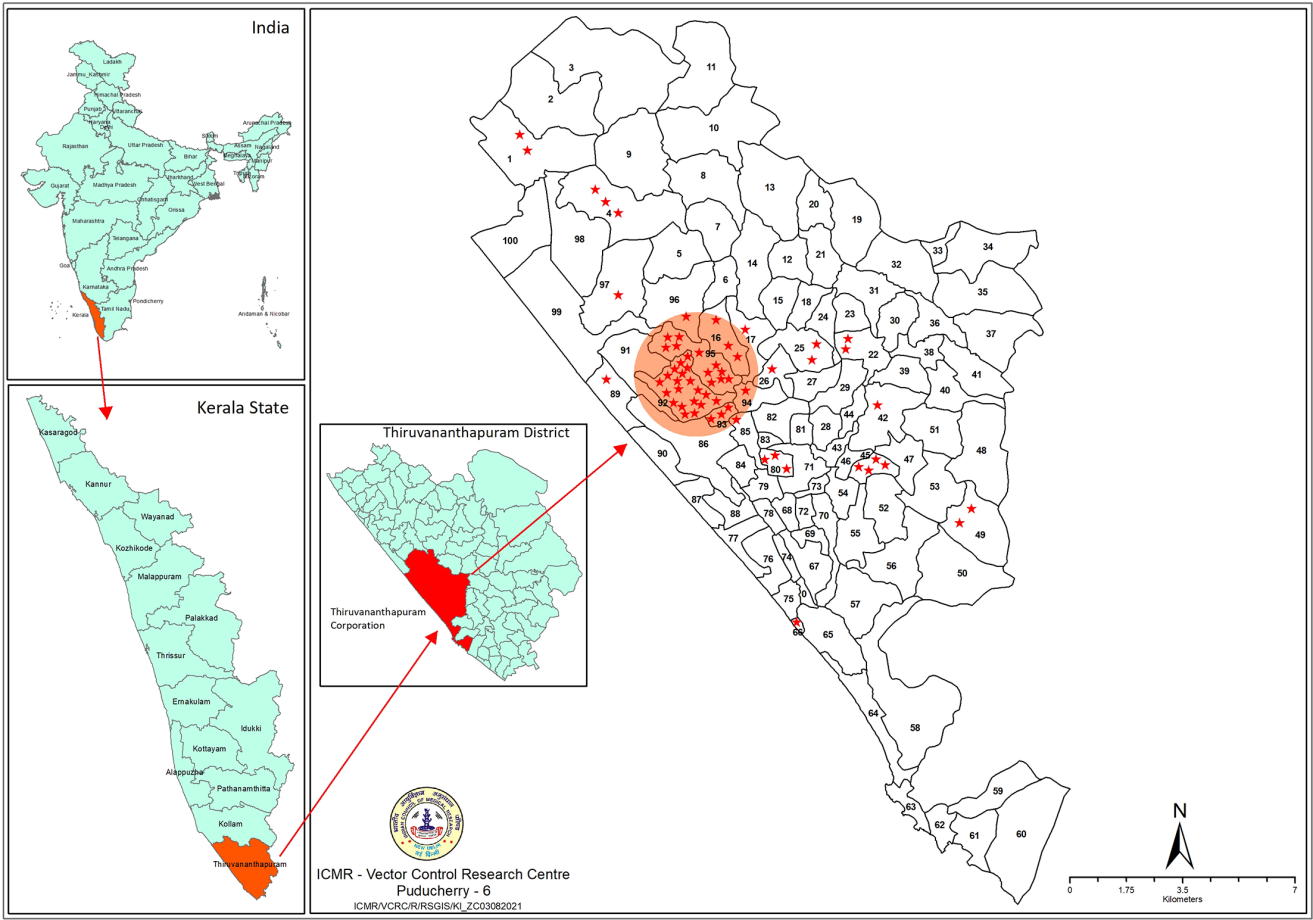
The study area (Thiruvananthapuram City Corporation) map and the distribution of the location of ZIKV-positive human cases recorded during 2021.

Thiruvananthapuram Municipal Corporation (referred to as city hereafter), the administrative capital of Kerala State, is located in the central coastal region in the southernmost Thiruvananthapuram District of Kerala, India [12]. It is the largest city of Kerala State, with an area of 214.86 sq. km and a population of 0.96 million (National Census of India, 2011). The city is administratively divided into 100 wards (Fig. 1) and is thickly populated with a population density of 4,457 sq. km^−1^. It has good connectivity to other parts of the country as well as globally. This city is also the administrative hub of Thiruvananthapuram District, which is bordered by the Arabian Sea on the western side, the Western Ghats on the eastern side and Nagercoil District of Tamil Nadu State, in the south.

Kerala State enjoys a tropical climate with about 3,000 mm rainfall, contributed by two monsoons (southwest and northeast). Also, intermittent showers are recorded during the summer season. Even though the total rainfall exceeded in the state during the year 2021 (23.4% above normal), causing massive floods in the central districts, this irregularity was not recorded in Thiruvananthapuram District.

### Epidemiological surveys

The first case of Zika fever was originally reported in a 24-year-old pregnant female, who was admitted to the Kerala Institute of Medical Sciences (KIMS), Thiruvananthapuram, on 28 June 2021. She developed fever, body pain and erythematous rashes whilst staying in a house near the hospital for her regular antenatal check-ups. Upon experiencing these symptoms, she was admitted to the hospital, and blood samples were taken for diagnosis. The KIMS Diagnostic Centre in Coimbatore suspected ZIKV infection in the patient, using real-time reverse transcription PCR (rRT-PCR), on 7 July 2021. Further confirmation of the infection was carried out at the Indian Council of Medical Research - National Institute of Virology.

We investigated the localities where the patient resided, prior to her hospital admission (her hometown of Nagercoil) and where she stayed towards antenatal care and developed the symptoms (near the hospital in Nanthancode). Also, we made inquiries with the hospital authorities, the patient and her relatives, as well as the clinical personnel who attended her and perused the hospital records of the case histories of fever cases reported for the past 6 months.

On confirmation of this case to be Zika fever, previously collected samples from fever cases of unknown aetiology from the hospital were also tested for ZIKV infection. This led to the detection of 13 additional samples collected earlier (from 15 May to 21 May 2021) to be positive for ZIKV. Most of these specimens were from the nursing and auxiliary staff at KIMS Hospital. We collected the line list of cases reported by the state health authorities in the city and geographically mapped those using RS-GIS software (Fig. 1).

Following the standard protocols, we collected 2.0 ml of venous blood samples from four suspected cases who reported with fever in the area surveyed, which were transferred to sterile DNase/RNase-free Eppendorf tubes for further processing. Informed consents were obtained from all the patients towards diagnostics and genomic studies of the arbovirus involved in their infection, if any.

As the study was undertaken as an outbreak investigation, at the request of the Health Department of the Government of Kerala, India, institutional ethical approval was obtained retrospectively from the Human Ethics Committee of ICMR-Vector Control Research Centre, Puducherry, India, towards further ongoing investigations.

A primary report on the outbreak investigation was submitted to Research Square, and a preprint of the same is available [13].

### Entomological surveys

Initially, a cross-sectional entomological survey was carried out in the localities where the hospital (Anayara – ward 92) is situated and also where the patient stayed for a week [Nanthancode – ward 25 (Fig. 1)], following standard protocols. This consisted of outdoor resting, indoor resting and BG-Sentinel trap (Clarke, USA) adult collections and immature (both larvae and pupae) surveys, for sampling mosquito populations.

Entomological surveys were further continued for a period of 3 months on fortnightly intervals. Both adult and immature mosquito surveys were carried out in both localities. Six-man-hour indoor resting and outdoor resting collections were performed in the urban areas. The detailed methodology followed for the entomological surveys is provided in our earlier investigations on ZIKV vector surveillance [11].

All the adult specimens collected were identified using standard taxonomic keys. They were then pooled based on species, sex and the abdominal conditions (unfed, full-fed and semi-gravid/gravid) in the case of females. The pooled specimens were transferred to sterile DNase/RNase-free Eppendorf tubes with 500 µl of TRI Reagent (Molecular Research Center, Inc., USA), stored at 4 °C and were transported to the laboratory for further processing. The immature specimens (larvae and pupae) were brought to the laboratory and were reared in ambient conditions. The emerged mosquitoes were taxonomically identified and pooled for further molecular diagnostics [11].

### Molecular diagnosis

Human samples collected were also transported to the laboratory maintaining cold chain conditions at 4 °C. Viral RNA extraction from the serum samples of these isolates was performed using the QiaAmp viral RNA kit (QIAGEN, Germany) following the manufacturer’s protocol. The mosquito pools were subjected to viral RNA isolation using the methodology described elsewhere [11]. The viral RNA isolates of both human as well as mosquito samples were then subjected to diagnostic rRT-PCR using the RealStar Zika Virus RT-PCR Kit (Altona Diagnostics, Germany), approved by FDA, USA, for EUA. Also, this kit had been evaluated for its high sensitivity (91%) and specificity (97%) for ZIKV detection [11]. All positive case samples recorded were further processed towards another set of non-structural protein 5 (NS-5) primers highly specific and sensitive for ZIKV [14], and the amplified short fragments were subjected to similarity analysis using NCBI-blast, towards confirmation.

The minimum infection rates (MIRs) for ZIKV for different vector species were calculated using the formula [number of positive pools/total number of specimens tested]×1,000, which assumes that a positive pool contains at least one infected mosquito [15].

In addition, we custom-designed three sets of DNA primers towards PCR amplification of the capsid–pre-membrane (C-prM) gene, envelope (E) gene and NS-5 gene of the ZIKV genome, which span most of the crucial and evolutionary significant mutations recorded in the virus genome [16]. The details of the primers designed by us, using Primer 3 software [17], were custom-synthesized (Metabion, Germany) and are given in Table 1.

**Table 1.** DNA primers synthesized and used in the study

Gene	Forward primers	Reverse primers
C-prM	AATTGTTGGCCTCCTGCTGA	TTACGGTGACACAACCTCGA
Envelope	GATAAACTTAGATTGAAGGGCGTGT	GGGGAGTCAGGATGGTACTT
NS-5	AGCCCTATGGAAAGGTCATTGAT	GACAACCCCGTTTATTAGAGAGG

Representative samples amongst the positive isolates were subjected to these three sets of primers, so as to amplify the desired gene sequences of ZIKV using the Transcriptor One-Step RT-PCR Kit (Roche, Germany), following the manufacturer’s protocol. The amplified DNA fragments were custom-sequenced by Agrigenome (India) using automated Sanger sequencing methodology and were subjected to genetic analysis using mega 11.0 [18].

### Molecular data analysis

The DNA sequences of CprM, E and NS-5 genes generated were aligned with representative sequences in the GenBank, available for different lineages of ZIKV, so as to delineate the SNPs as well as the deduced synonymous and non-synonymous (NS) mutations amongst the isolates collected from Thiruvananthapuram.

Also, phylogenetic analysis of these nucleotide sequences was inferred by using the maximum likelihood method and the Tamura–Nei model [18,19]. The tree with the highest log-likelihood was deduced. The percentage of trees in which the associated taxa clustered together is shown next to the branches. Initial trees by the heuristic search were obtained automatically by applying the neighbour-join and BioNJ algorithms to a matrix of pairwise distances estimated for nucleotides using the Tamura–Nei model and then selecting the topology with the superior log-likelihood value. The trees were drawn to scale, with branch lengths measured in the number of substitutions per site (next to the branches). This analysis involved 15 nucleotide sequences.

## Results

### Epidemiological investigations

As described earlier [13], a clustering of infections was recorded in the Anayara village where the hospital is located. All the hospital staff who were found infected with ZIKV were residing in localities near the hospital. Besides, the presumed index case reported (24/F) could not be the actual index case. She temporarily resided in the Nanthancode ward of the city and frequently visited the KIMS Hospital for periodical antenatal investigations prior to her ZIKV disease episode. The symptoms reported by this patient were described as fever, rashes, conjunctivitis, muscle and joint pain, malaise and headache by the medical officer who attended her. She delivered a normal baby and was discharged from the hospital on 6 July 2022. Her confirmed diagnosis of Zika fever led to the testing of several other suspected fever cases sent earlier for diagnosis, which turned out to be ZIKV positives and thereby unveiled the outbreak. The earliest fever case samples sent for diagnostics on 15 May 2021 were from two nursing staff of the hospital, who were confirmed as having ZIKV infection on re-testing these retrospectively. Thus, we infer that the unknown index case would have visited the hospital during the first week of May 2021, as per hospital records. He/she would have been hospitalized and would have been discharged from the hospital undiagnosed as a ZIKV-infected patient. Many non-resident Indians visit as patients in this hospital as per their records. As the hospital itself was found vulnerable, the infection would have spread amongst the nursing and accessory staff of the hospital, as a general fever, which went undiagnosed for a period of about 2 months, until the presumed index case was reported. Altogether, a total of 83 confirmed cases of Zika fever were reported during 2021 in the city as per the government Integrated Disease Surveillance Programme, and all were geographically mapped in Fig. 1.

We have also processed four suspected human samples, out of which two samples were detected to be positive for ZIKV infection. The positive cases had typical presenting symptoms of fever with rash, muscle pain, joint pain, conjunctivitis and general tiredness. The onset of symptoms was abrupt, and the fever lasted for only 1 day followed by the disappearance of the rash within 2–3 days. The patient was administered symptomatic treatment by the hospitals they attended to and had recovered within 1 week of the onset of symptoms.

### Entomological studies

**Fig. 2.**
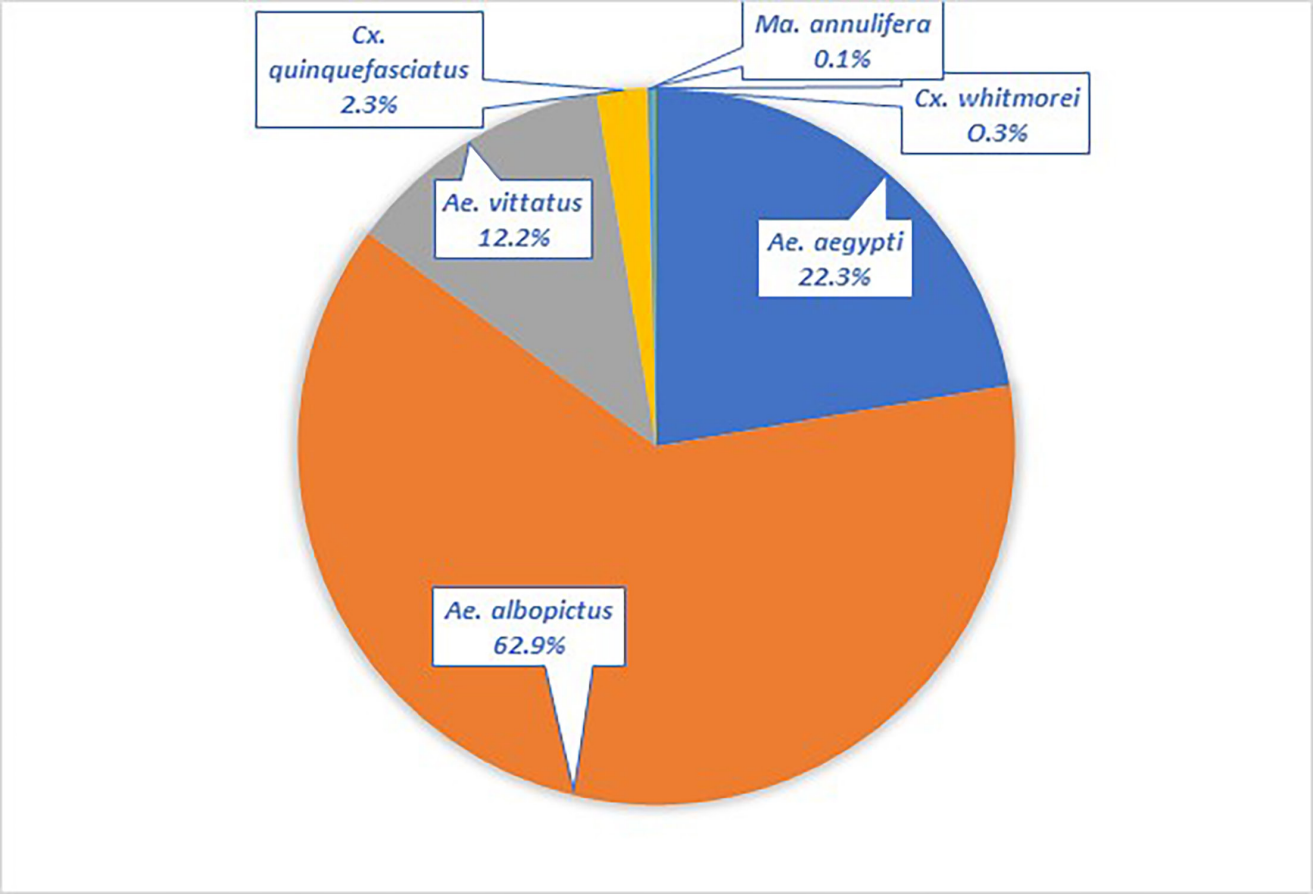
Species composition of mosquitoes collected during the Zika fever outbreak investigation in Thiruvananthapuram City, Kerala, India, during July–September 2021.

On the initial assessment of cross-sectional entomological surveys, it was found that both the hospital premises, as well as neighbouring areas, harboured profuse breeding sources of *Aedes* mosquitoes. Entomological surveys carried out fortnightly for a period of 3 months yielded a total of 2,277 specimens of adults as well as immature (larvae and pupae) specimens of *Culex quinquefasciatus*, *Culex whitmorei*, *Mansonia annulifera*, *Ae. aegypti*, *Ae. albopictus* and *Aedes vittatus* (Fig. 2). The predominant species collected in the investigations was found to be *Ae. albopictus* (62.88%). The species composition of the specimens collected and processed and the number of pools found positive for ZIKV by rRT-PCR during the outbreak period are provided in Table 2.

**Table 2.** Details of entomological collections carried out and processed for infection status by rRT-PCR for ZIKV infection from Thiruvananthapuram District of Kerala State, India, during 2021

Species	No. of mosquitoes collected			No. of mosquitoes processed	No. of pools processed	Pools positive/no. of mosquitoes	
	Female	Male	Total			Female	Male
Field-collected adult specimens							
*Ae. vittatus*	17	0	17	11	4	1	0
*Cx. quinquefasciatus*	45	1	46	46	8	0	0
*Ma. annulifera*	3	0	3	0	0	0	0
*Ae. aegypti*	44	31	75	72	33	1	2
*Ae. albopictus*	281	200	481	464	118	9	5
Total	**390**	**232**	**622**	**593**	**163**	**11**	**7**
Adult emergence from immature collections							
*Ae. vittatus*	101	159	260	239	20	0	0
*Cx. whitmorei*	6	0	6	0	0	0	0
*Cx. quinquefasciatus*	0	6	6	0	0	0	0
*Ae. aegypti*	234	198	432	310	33	2	0
*Ae. albopictus*	446	505	951	370	53	0	1
Total	**787**	**868**	**1,655**	**919**	**106**	**2**	**1**
**Grand total**	**1,177**	**1,100**	**2,277**	**1,512**	**269**	**13**	**8**

Altogether, 1,466 *Aedes* specimens were processed for ZIKV infection in 261 pools (species-wise) by rRT-PCR protocols described elsewhere [11]. Amongst these, 19 pools were found positive for ZIKV infection by real-time PCR, which included 15 pools of *Ae. albopictus*, 3 pools of *Ae. aegypti* and 1 pool of *Ae. vittatus*. Field-collected adult specimens (*n*=17) as well as emerging adult specimens from immature collections (*n*=2) were found to be infected with ZIKV. Amongst the eight ZIKV-positive field-collected *Ae. albopictus* female pools (one positive pool was from the emergence lot), seven were unfed, and one was a semi-gravid specimen. Also, a lone field-collected female pool of *Ae. aegypti* was found positive (another positive pool was from the emergence lot) and constituted of unfed specimens. These observations clearly indicate the vectorial role of these two species in the outbreak, and this is the first report on the role of *Ae. albopictus* in the transmission of ZIKV from India. In addition, ZIKV was also detected from a field-collected pool of *Ae. vittatus*. However, this detection comes from a pool of fully fed adult specimens. As the infection picked up by the reaction could also have been from specimens’ blood meals, the role of this species as a vector cannot be ascertained. Furthermore, male specimens from both species (*Ae. albopictus* – six pools and *Ae. aegypti* – two pools) from adult emergence as well as field-collected specimens and female unfed pools from adult emergence (*Ae. aegypti* – two pools) were found infected with ZIKV infection (Table 2), indicating trans-ovarial transmission of the virus during the outbreak.

The MIRs estimated for *Ae. albopictus*, *Ae. aegypti* and *Ae. vittatus* specimens were 17.9, 7.8 and 3.6, respectively. None of the 47 *Cx*. *quinquefasciatus* specimens processed for arboviral infections was found positive for infection with ZIKV (Table 2). The adaptation of the virus to *Ae. albopictus* in the current investigation is also intriguing, as the maximum number of infections was detected in *Ae. albopictus* specimens.

### Genetic characterization of the virus

All custom-sequenced nucleotide sequences were submitted to GenBank, and the respective accession numbers of ZIKV gene sequences detected from the positive human as well as mosquito isolates are provided in Table 3. The overview of the synonymous and NS mutations delineated, compared with other lineages of ZIKV, is enlisted in Table 4.

**Table 3.** Details of GenBank accession numbers of ZIKV gene fragments generated in the study

Genetic markers	Diagnostic markers						CprM		Envelope		NS-5	
Host species	*Homo sapiens*		*Ae. aegypti*	*Ae. albopictus*			Human	*Ae. albopictus*	Human	*Ae. albopictus*	Human	*Ae. albopictus*
Sample details	24F	40F	Field-collected male	Emerged male	Field-collected male	Field-collected female						
GenBank acc. no.	MZ670001	MZ686204	OP947010	OP947011	MZ686203	MZ686202	OP679002	OP679003	OP679000	OP679001	OP678998	OP678999

**Table 4.** The NS mutations recorded in the genome of different lineages of ZIKV

Different lineages	Genome regions/amino acid positions where mutations were recorded										
	Capsid	Pre-membrane				Envelope			NS-5		
	106	1	17	22	160	348	470	473	114	267	322
**African lineage**											
Uganda MR 766 1979	A	A	S	A	I	D	T	V	M	A	V
**Asian lineage**											
*Aedes* species P6-740 Malaysia	A	V	S	A	I	D	T	V	T	A	I
*H. sapiens* BKK04 Thailand 2016	A	A	S	A	I	D	T	M	M	V	I
*H. sapiens* MU-DMSC4 Thailand 2016	T	A	S	A	I	D	T	M	M	V	I
*H. sapiens* China YN001 2016	T	A	S	A	I	D	T	M	M	V	I
*H. sapiens* Thailand 2006	A	V	S	A	I	D	T	V	M	V	I
**American lineage**											
*H. sapiens* PE243 Brazil 2015	A	A	N	A	I	D	T	M	V	V	I
*H. sapiens* Venezuela 2016	A	A	N	A	I	D	T	M	V	V	I
*H. sapiens* Puerto Rica 2016	A	A	N	A	I	D	T	M	V	A	I
*H. sapiens* Brazil 2015	A	A	N	A	I	D	T	M	V	V	I
**Indian lineage**											
*H. sapiens* 20366 Rajasthan 2018	T	A	S	A	M	D	T	M	M	A	V
*H. sapiens* 17844 Rajasthan 2018	T	A	S	A	M	D	T	M	M	A	V
*H. sapiens* H-8900 Maharashtra 2021	T	A	S	A	M	N	A	L	M	V	V
*H. sapiens* Thiruvananthapuram 2021	T	A	S	T	M	N	A	L	M	V	I
*Ae. albopictus* Thiruvananthapuram 2021	T	A	S	T	M	N	A	L	M	V	I

#### Capsid–pre-membrane gene

The C-prM [corresponding to 107–2,488 bp sequences of the MR766 reference genome (GenBank acc. no. MW143021)] region amplified was analysed for phylogenetically important NS mutations, such as 'T106A' in the capsid and 'V1A' and 'S17N' in the pre-membrane (prM) regions, which have been observed in other ZIKV lineages [14]. Thiruvananthapuram isolates had threonine at the one hundred sixth position of the capsid gene ('A106T'), consistent with the ancestral Asian lineage and alanine at the first position ('V1A') of the prM gene, similar to the American lineage. However, one of the critical mutations associated with microcephaly in newborns [20], 'S17N' in the prM gene, was not prevalent in the Indian lineage. Instead, serine was present at the seventeenth position, consistent with the ancestral Asian lineage. Additionally, two more NS mutations, 'A22T' and 'I160M', were also elucidated in these isolates (Table 3). The 'A22T' mutation was unique to our isolates, whilst the 'I160M' mutation had previously been reported in Jaipur isolates from 2018 [10].

#### Envelope gene

The E gene was analysed for a major mutation, ‘V473M’, which was reported to be the major contributing mutational change which enabled the increase in virulence of the virus [21]. However, in our isolates, we recorded a novel alternative mutation from valine to leucine (‘V473L’). Also, the E gene had two more NS mutations, viz. ‘D348N’ and ‘T470A’. The former had not been recorded either in the Asian lineage or the American lineage of the virus, till date. The isolate (OM666892) sampled in 2021 from Maharashtra also recorded this unique mutation (‘V473L’ and ‘T470A’). Thus, amongst the three NS mutations recorded in 2021 in Indian isolates, two (‘V473L’ and ‘D348N’) mutations were found to be unique to our isolates (Table 3).

#### Non-structural protein 5 gene

For elucidating the crucial mutation recorded in the American lineage of ZIKV (‘M114V’), which resulted in the divergence of the American lineage from the Asian lineage [22], the region encompassing this region of the NS-5 gene was analysed, one each for humans as well as *Ae. albopictus*. Both these isolates had methionine in the one hundred fourteenth position of the NS-5 gene, similar to the ancestral Asian lineage [23].

### Phylogenetic analysis

**Fig. 3.**
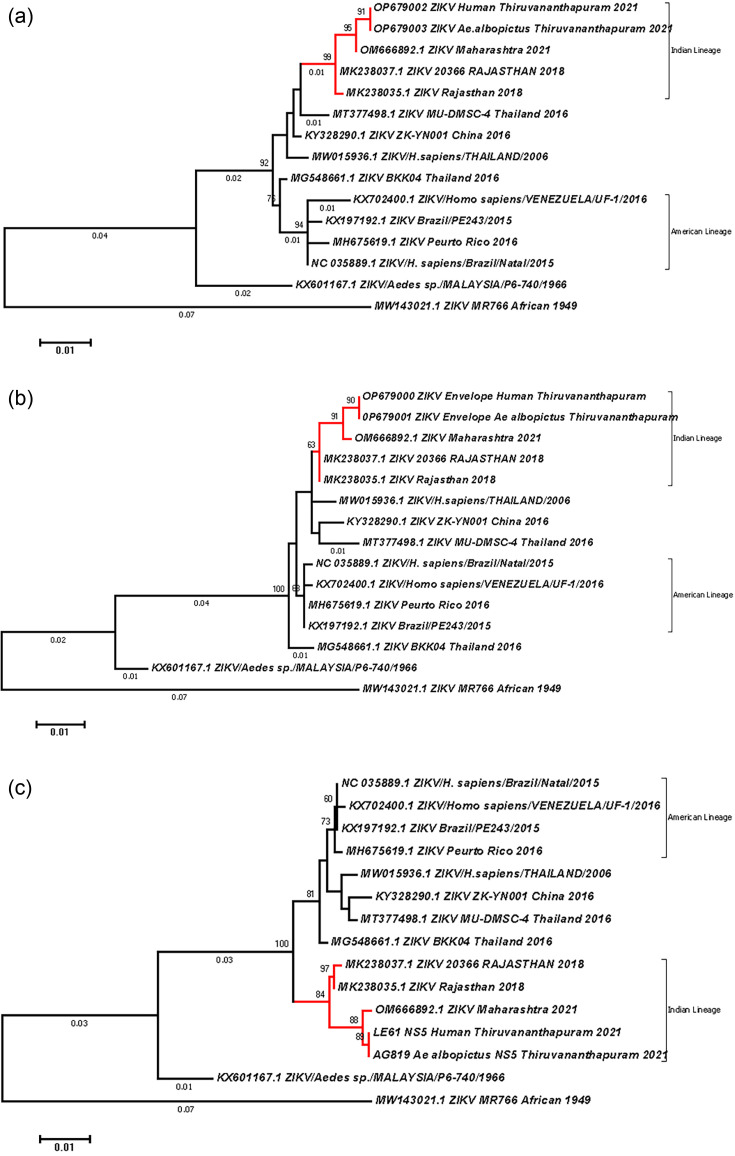
Phylogenetic analysis of (**a**) C-prM, (**b**) E and (**c**) -NS-5 gene sequences of ZIKV isolates from Thiruvananthapuram. Samples included in the Indian lineage are highlighted.

Altogether, 734, 615 and 680 consensus base pair nucleotide sequences from the C-prM, E and NS-5 gene regions, respectively, were subjected to phylogenetic analysis using the maximum likelihood method and the Tamura-Nei model. The resulting phylogenetic trees, which had the highest log-likelihood values, are presented in Fig. 3(a–c).

The phylogenetic trees generated using the three genetic markers investigated revealed a well-segregated Indian clade (highly significant bootstrap support values of 92.0 for C-prM, 100.0 for E and 99.0 for NS-5), distinct from that of the ancestral Asian lineage as well as the American clade of ZIKV (Fig. 3). This trend clearly evinces the emergence of a novel Indian lineage of ZIKV derived from the ancestral Asian lineage.

**Fig. 4.**
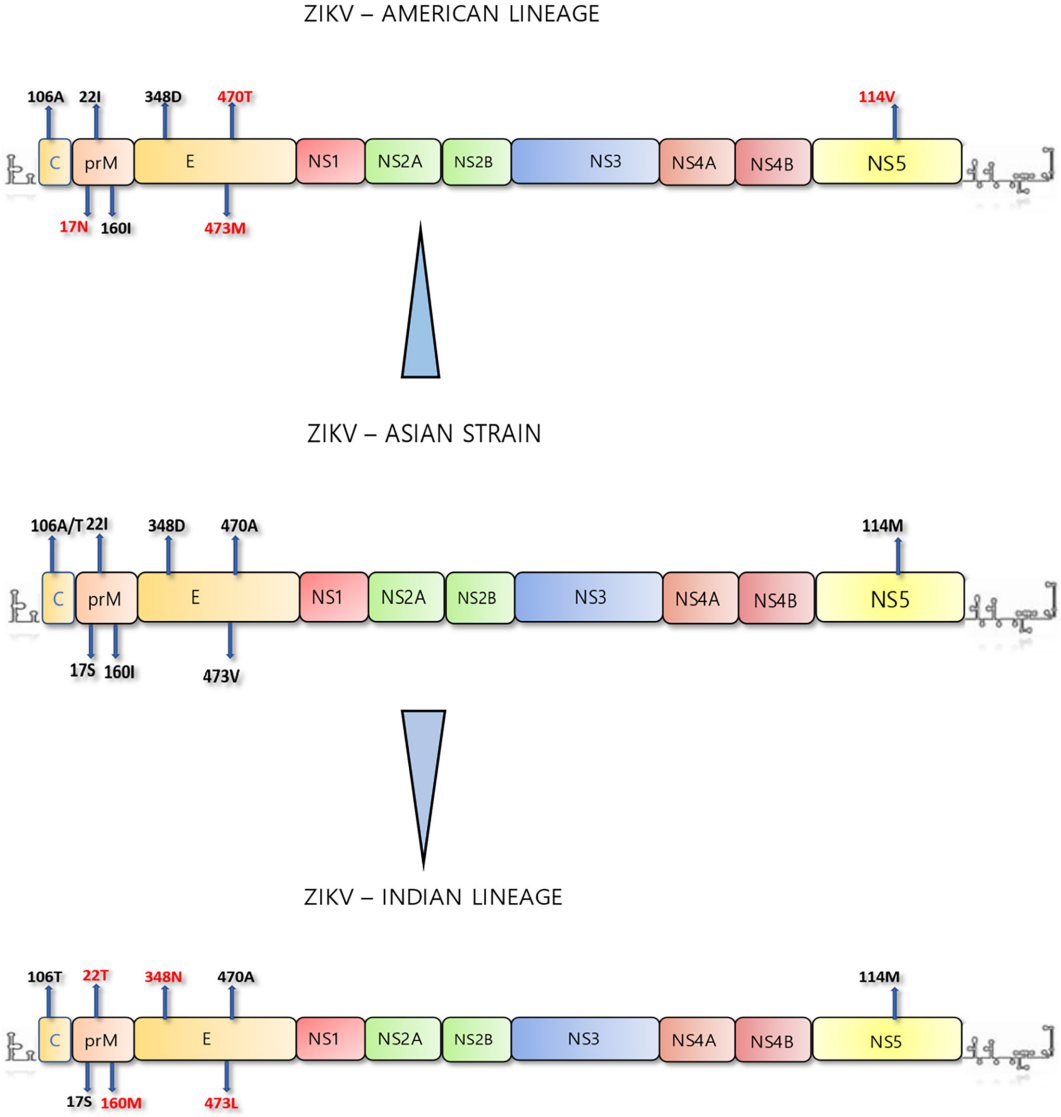
Graphical representation of the genome structural analysis of ZIKV of different lineages of the virus. Unique NS mutations are shown in red.

A graphical representation of NS mutations identified in the study, comparing the ancestral Asian genotype, the American lineage and the current Indian lineage, is shown in Fig. 4. Altogether, five unique mutations were identified in the C-prM, E and NS-5 gene fragments of the recent Indian isolates. These findings also strongly support the genetic divergence of the Indian isolates from previously reported ZIKV lineages, highlighting the evolutionary trends of the virus in the Indian context.

## Discussions

Sporadic outbreaks of Zika fever had been reported from Gujarat and Tamil Nadu in 2017 and Rajasthan and Madhya Pradesh in 2018 before the occurrence of the reported outbreak of ZIKV in Thiruvananthapuram, Kerala. Also, subsequent outbreaks had been reported from India in Maharashtra (July 2021) [24] and in Uttar Pradesh (October–November 2021) [24]. A recent study [25] reported sporadic records of ZIKV-positive cases from different states of India (number of cases given in parenthesis), viz. Punjab (one), New Delhi (one), Rajasthan (one), Jharkhand (one) and Telangana (one). Furthermore, during 2023, Zika infections were also reported from Maharashtra, Karnataka and Kerala [26]. Altogether, ZIKV infections have been detected from 17 states/union territories of the country so far. These observations indicate that there has been an increase in the incidence of ZIKV infections in the country, post-2018.

An earlier report [27] proposed two lineages of ZIKV in India. This was based on the variations recorded in the partial E gene sequences (MF173410) of an isolate from Gujarat, whose sequences do not match with any known Asian isolates of the virus, and the authors cited it as a primitive Asian lineage of the virus. However, in the present investigation, we did not record any such occurrence of such a primitive lineage of the virus closely linked to the ancestral lineage of the Asian genotype, during the current outbreak (Fig. 3).

Our investigations looked into the evolutionary trends of the virus emphasizing the reported crucial mutations of the ZIKV genome, in the wake of the Zika fever outbreak in Thiruvananthapuram during 2021. The crucial mutation ‘V473M’ was reported to be the major contributing NS mutation which enabled an increase in virulence and was cited to be responsible for the recent emergence of Zika fever during 2015, causing severe disease manifestations [14]. Also, a high level of conservation in the E gene sequences was reported in the American lineage of the virus which caused the massive outbreak in South American countries [21]. Valine in the ‘473’ position of the E gene, recorded amongst the earlier Asian genotypes, underwent an NS change to methionine during the PHEIC of Zika fever recorded during 2015–2016. However, amongst all the isolates collected across the country during 2021 (our isolates as well as the isolates from Maharashtra), another novel and alternative NS 'V473L' variant was consistently observed. ‘V473M’, along with other mutations, had also been implicated towards an increase in the epidemic potential in urban transmission by *Ae. aegypti* [28]. In the Thiruvananthapuram outbreak, this mutation was not reverted back but was substituted by another, ‘V473L’. Does this have any implication on the change of the major vector species to *Ae. albopictus*? This remains an intriguing concept to be investigated. In addition, two unique mutations, ‘D348N’ and ‘T470A’, were also recorded from these Indian isolates. The bootstrap values recorded amongst the American and the Indian lineages based on the E gene sequences were also highly significant (100.0). Also, the crucial distinguishing mutation ‘M114V’ universally reported amongst the isolates in the NS-5 gene of the American lineage [21], which had been attributed to the epidemic of the virus in the American region, was absent in the Indian isolates. This NS mutation is located in the crucial methyl transferase domain which interacts with the RNA-dependent RNA polymerase domain of the NS-5 gene. The phylogenetic analysis based on NS-5 gene sequences also showed a segregation of the Indian lineage clade with a bootstrap value of 99.0 (Fig. 3c). The analysis of the amplified C-prM region revealed two mutations. Capsid ‘T106A’ reported in the American lineage is not recorded in the Indian lineage. Two other mutations, 'A22T' and 'I160M', were recorded in Indian strains. The 'A22T' mutation was uniquely identified in the isolates from this study, whilst 'I160M' is specific to Indian isolates and had been previously reported in other Indian samples also. Notably, the critical mutation 'S17N' contributing to foetal microcephaly was absent in our isolates. The bootstrap values in the phylogenetic tree deduced for the C-prM gene were also found to be highly significant (92.0%), for the Indian clade which included Kerala and Maharashtra isolates, post-2018.

Thus, the phylogenetic analysis of these three important genetic markers clearly evinced that a different lineage of ZIKV is re-emerging in India (Fig. 3a–c). Five unique mutations were also recorded in these study isolates not reported in the American lineage or the ancestral Asian strain of the virus.

Both *Ae. aegypti* and *Ae. albopictus* have a wide distribution in India [11]. The regular intermittent rainfalls during the summer season in Kerala State result in the build-up of *Aedes* population in the city, as described in our previous investigations, and the introduction of a novel lineage of virus into an immunologically naïve population mostly leads to arbovirus outbreak situations as described elsewhere [12]. The entomological investigations carried out recorded abundant populations of *Aedes* species in the region and the natural infection of ZIKV in all the *Aedes* species prevalent, viz. *Ae. aegypti*, *Ae. albopictus* and in a lone pool of *Ae. vittatus* in the study area. The maximum infection rate was recorded in *Ae. albopictus*, which was not recorded as a vector species in India in either of our previous investigations [11] or elsewhere in the country. However, this species had been reported as the vector species in Gabon [5]. Also, in our investigations, male specimens of *Ae. albopictus Ae. aegypti* were found positive for natural infection indicating trans-ovarian transmission of the virus. Similar observations on *Ae. aegypti* had been reported elsewhere [29].

## Conclusion

The NS mutations recorded amongst Indian isolates, viz. ‘A106T’ (capsid); ‘V1A’, ‘A22T’ and ‘I160M (prM); and ‘D348N’, ‘T470A’, ‘V473L’ (E), ‘V267A’ and ‘I322V’ (NS-5), are highly significant and point towards the evolutionary trends of ZIKV in the Indian context. These mutations indicate the ongoing evolutionary adaptations of the virus within this region, potentially contributing to its unique lineage characteristics. Whilst the American lineage of ZIKV had the following signatory NS mutations – C: ‘T106A’; prM: ‘S17N’; E: ‘V473M’; and NS-5: ‘M114V’ [14], the emerging Indian lineage recorded five unique NS mutations, viz. prM: ‘A22T’ and ‘I160M’ and E: ‘D348N’, ‘T470A’ and ‘V473L’ (Fig. 4). To summarize, this study looked into the C-prM, E and NS-5 gene sequences, spanning the evolutionary significant regions of the ZIKV genome from this and other recent outbreaks in the country. The genetic analysis of these crucial genes of ZIKV indicated the emergence of a unique Indian lineage (Fig. 3). A similar molecular evolutionary situation occurred during the 2007–2011 outbreak of Chikungunya virus, owing to a mutation in the E gene (A226V). The virus caused havoc in Kerala and the neighbouring Karnataka states in India, where *Ae. albopictus* is the predominant *Aedes* species [30,31].

The reports on our investigations were duly disseminated to the Department of Health Services, Govt. of Kerala, this being a collaborative venture as requested by them. The suggestions put forward were readily accepted by the government machinery, and very intensive immature control operations (source reduction) as well as adulticidal insecticide applications were resorted to, for a period of 3 months by them, in the city limits concentrating on the foci of transmission, as advised by us based on our random entomological surveys. There was a drastic reduction in the *Aedes* population in the city, and no Zika fever cases were reported where cases were reported post November 2021.

The emerging ‘Indian lineage’ of ZIKV may have the potential to evolve as the predominant strain, leading to unprecedented outbreaks of the disease in the future. Hence, investigations on the implications of the altered gene expression of the described mutations, including the adaptation of the virus to *Ae. albopictus*, the predominant *Aedes* species in many states of the country, remain inevitable to forecast and prevent more future outbreaks in India. A systematic vector surveillance programme on arboviral diseases in the country remains imminent to forecast and prevent the increasing incidence of *Aedes*-borne viral diseases in the country.

### Limitations of the study

Only six samples of the virus isolates could be amplified and sequenced by conventional diagnostic PCR, though 21 positives (2 human and 19 real-time ZIKV-positive samples) were obtained in the real-time PCR analysis in the investigation. This may be owing to the lower sensitivity of the conventional RT-PCR in detecting the low threshold load of the virus in mosquito samples detected by real-time PCR. We analysed the sequences of four genes in the genome of the virus which had been reported to be of high evolutionary significance in previous studies, instead of carrying out a whole-genome analysis, owing to the economic constraints. Although *Ae. albopictus* played a significant role in this outbreak, towards incrimination of the species as a vector of the virus in India, further studies on vector competence are warranted. In the present study, we are documenting the natural infection of the virus in this mosquito species for the first time in India.

## Abbreviations

C-prM: capsid-pre-membrane
E: envelope
KIMS: Kerala Institute of Medical Sciences
NS-5: non-structural protein 5
NS: non-synonymous
PHEIC: Public Health Emergency of International Concern
prM: pre-membrane
rRT-PCR: real-time reverse transcription PCR
WHO: World Health Organization
ZIKV: Zika virus
